# Development and validation of a short form psychometric tool assessing the caregiving Challenge of Living with Cystic Fibrosis (CLCF-SF) in a child

**DOI:** 10.1080/08870446.2023.2231489

**Published:** 2023-07-05

**Authors:** Gareth McCray, Holly F. Hope, Claire Glasscoe, Jonathan Hill, Alexandra Quittner, Kevin W. Southern, Gillian A. Lancaster

**Affiliations:** a School of Medicine, Keele University, Keele, UK; b Division of Psychology and Mental Health, Centre for Women’s Mental Health, University of Manchester, Manchester, UK; c Department of Women’s and Children’s Health, Institute of Translational Medicine, University of Liverpool, Alder Hey Children’s NHS Foundation Trust, Liverpool, UK; d School of Psychology and Clinical Language Sciences, Reading University, Reading, UK; e Behavioral Health Systems Research, Miami, FL, USA

**Keywords:** Caregiver, child, cystic fibrosis, genetic algorithm, short-form, treatment burden

## Abstract

**Objective:**

Caring for a child with cystic fibrosis (CF) is a rigorous daily commitment for caregivers and treatment burden is a major concern. We aimed to develop and validate a short form version of a 46-item tool assessing the Challenge of Living with Cystic Fibrosis (CLCF) for clinical or research use.

**Design:**

A novel genetic algorithm based on ‘evolving’ a subset of items from a pre-specified set of criteria, was applied to optimise the tool, using data from 135 families.

**Main outcome measures:**

Internal reliability and validity were assessed; the latter compared scores to validated tests of parental well-being, markers of treatment burden, and disease severity.

**Results:**

The 15-item CLCF-SF demonstrated very good internal consistency [Cronbach’s alpha 0.82 (95%CI 0.78–0.87)]. Scores for convergent validity correlated with the Beck Depression Inventory (Rho = 0.48), State Trait Anxiety Inventory (STAI-State, Rho = 0.41; STAI-Trait, Rho = 0.43), Cystic Fibrosis Questionnaire-Revised, lung function (Rho = −0.37), caregiver treatment management (*r* = 0.48) and child treatment management (*r* = 0.45), and discriminated between unwell and well children with CF (Mean Difference 5.5, 95%CI 2.5–8.5, *p* < 0.001), and recent or no hospital admission (MD 3.6, 95%CI 0.25–6.95, *p* = 0.039).

**Conclusion:**

The CLCF-SF provides a robust 15-item tool for assessing the challenge of living with a child with CF.

## 1. Introduction

Cystic Fibrosis (CF) is a chronic, life-shortening, genetically transmitted condition usually diagnosed in infancy, and treatment regimens focus on pro-active management of health *via* a diverse range of interventions (Davies et al., 2007). These interventions require a considerable time investment by patients and families alike (Sawicki et al., 2013). Clinicians have long continued to express concern over the cumulative burden the treatment protocols place on both those affected with CF and their families (Jones et al., 2002; Ziaian et al., 2006). Qualitative and quantitative studies have similarly documented the demands on families of caring for a child with CF (Foster et al., 2001; Lowton, 2002; Modi & Quittner, 2006; Slatter et al., 2004). Most recently the James Lind Alliance Priority Setting Partnership, comprising patients, carers, and clinicians, set out the top 10 priorities in CF—number 1 being to simplify treatment burden (Rowbotham et al., 2018), emphasising that the concerns seen over many years are still pertinent today.

The key interventions for good CF outcomes have remained constant over many years and address the sequelae of the CF gene defect. Over the past decade, there has been considerable progress in the field with many people with CF now able to access therapies that correct the underlying molecular defect. These modulator therapies often have a profound impact on clinical outcomes. The European guidance outlines the implementation of modulator therapy and some of the challenges, including that other therapies should not be stopped unless as part of a clinical trial (Southern et al., 2023). The implementation of these therapies has resulted in some rationalization of the treatment burden, but the core requirements for therapy remain unchanged. There has been a ‘shifting of the goalposts’. Not all people with CF are eligible for modulator therapy and this has only recently become available for the younger age range (Southern et al., 2023).

As real-world experiences with CFTR modulators increase with time (Dagenais et al., 2021), interest in both its positive and negative extra-pulmonary effects is burgeoning (Mayer-Hamblett et al., 2023; Sergeev et al., 2020). Although quality of life may improve for many initiating CFTR modulators, a variety of negative side effects with potential impacts on safety and well-being have been reported, including neuropsychiatric changes (Spoletini et al., 2022; Talwalkar et al., 2017). Worsening mental health (recent case reports of suicide attempts in adolescents after starting Elexacaftor/Tezacaftor/Ivacaftor (ETI) therapy; Tindell et al., 2020; Arslan et al., 2023) and new onset mental health challenges have been reported (Heo et al., 2022; McKinzie et al., 2017). In a recent survey of CF providers in the United States (Bathgate et al., 2023), a substantial proportion of people with CF are experiencing side-effects (on average 20–24%), with the most common side-effects including new onset mood disorders (depression, anxiety), worsening of mental health, insomnia, cognitive fogging (memory, attention), and headaches.

The concern over the impact of the CF condition on children with CF and their families precipitated the development of disease-specific quality of life measures designed for children, adults, and caregivers (Gee et al., 2000; Boling et al., 2003; Quittner et al., 2005). Recent research has also called for a CF-specific caregiver burden measure, particularly in light of radical additions to the treatment protocol for CF (Brodlie et al., 2015; Quittner et al., 2014; Sawicki et al., 2013; Smith et al., 2010). With no method to assess the demands of increasingly complex interventions conducted at home upon the caregiver, trials of new interventions are being hampered (Davies et al., 2020; Rowbotham et al., 2018). The lack of a suitable measure prompted our previous work (Glasscoe et al., 2022), and the work described here, to create a long-form and short-form measure of the Challenge of Living with CF (CLCF) with its associated treatment burden.

The development of the CLCF raised important topics for caregivers and helped them develop questions they had for the clinical team (Glasscoe et al., 2022). However, it was evident that a short-form (SF) version was needed, particularly for research purposes and to evaluate the impact of new treatments for CF. Families described the original long form with 239 items (now abridged as a clinical questionnaire) as ‘burdensome’ to complete and on occasion affected them deeply prompting a response by the psychologist/psychosocial specialist member of the research team. An in-depth questionnaire does offer a clinical tool for a clinical psychologist/psychosocial specialist within the multidisciplinary team particularly in the context of an annual review and can be empowering for the family (Patel et al., 2011a, 2011b). However, a short form would be more practical, could be used in a research or clinical environment, without significant time demand. Furthermore, during a routine appointment where an SF might be completed, parents could complete it, hand it in, and have it scored and discussed in the same appointment, benefiting both families and the CF team supporting them. Within a research context, where families are likely expected to complete the measure multiple times the SF version again is preferable.

To create a unidimensional scale of a manageable length with sufficient domain coverage for parents/caregivers to use, a principled method of item reduction was required (Lancaster, 2009). In the area of measurement and scale construction, genetic algorithms (GAs) have been applied to several complex problems, including the creation of parallel test forms, i.e. tests using different sets of items with as close as possible psychometric characteristics (Sun et al., 2008), the construction of tests for cognitive diagnosis (Finkelman et al., 2009), and the assembly of computer-assisted tests (Hwang et al., 2005). GAs have also recently been applied to the production of short-form measures (Sahdra et al., 2016; Sandy et al., 2014; Yarkoni, 2010). A GA is a stochastic iterative technique, analogous to the action of evolution by natural selection that can be used for the optimisation of a set of parameters (Coley, 1999). It starts with a ‘population’ of possible randomly generated solutions to a particular question. In the case of SF creation, each member of the population would be a particular combination of items. A given number of population members would be randomly generated to initialize the algorithm. It then assesses the suitability of those answers according to some pre-defined criteria. For example, in SF creation, we might want to correlate the scores on the SF with the scores on the long form, and the higher the correlation the better (‘fitter’) a given member of the population. After this, the worst performing answers are ‘culled’ from the population and the best performing answers ‘breed’ (swap information). This process is iterated multiple times until the user stops the process, normally, when it seems like a stable answer has been reached. In SF creation, this might be when we fail to improve the fitness of the fittest member of the population (i.e. the best subset of items) for more than a hundred generations. This was the approach used in this study.

The main aim of the study was to develop a CLCF short-form (CLCF-SF) to improve the utility of the instrument as a screening tool in clinical practice and for use as an outcome measure in clinical trials of new interventions. A secondary aim was to provide evidence for the reliability and validity of the CLCF-SF. Specific research questions were:

- Which items from the full CLCF can be combined in a measure, with good psychometric properties, to best assess how well caregivers are managing the challenge of caring for a child with CF?
- How reliable is the CLCF-SF and its component items?
- How does the CLCF-SF score relate to other measures with which it is expected to correlate (i.e. concurrent validity)?

We expected that the CLCF-SF would correlate positively both with the Beck Depression Inventory (Beck et al., 1996) and the State-Trait Anxiety Inventory (Spielberger et al., 1983), because it was hypothesised that the higher the perceived challenge of caring for a child with CF might be, the more pronounced the levels of depression and anxiety may become. We also expected that a low lung function score calculated from the CF questionnaire (Quittner et al., 2005), indicating poor respiratory condition, would correlate negatively with the CLCF-SF. The CLCF-SF scores were also expected to correlate positively with two derived measures of treatment management and a sum score of the number of treatments a child was on, a higher score indicating a higher level of perceived difficulty.

- How do the scores on the CLCF-SF discriminate between groups that are expected to have different levels of the challenge of living with a child with CF? (i.e. discriminant validity). We hypothesised that unwell children at the time of data collection compared to well children with CF would have a higher CLCF-SF score on average, and similarly for those children with CF who had had a recent hospital admission compared to those children who had not been admitted to hospital.

## 2. Methods

### 2.1. Participants

Data were collected between 2008 and 2010 from 135 caregivers with at least one child aged up to 14 years with a confirmed diagnosis of CF. The data were taken from three inter-linked research studies led by the same clinical investigators (KW, CG) and conducted from the main study site in Liverpool: (i) CLCF validation study (October 2008 to July 2009), (ii) sensitivity to clinical change study (October 2008 to May 2009; Dyer, 2010), and (iii) an NIHR funded Home Intravenous Antibiotic Treatment (HIVAT) study (March 2009 to September 2010) (ISRCTN65724841) for which the CLCF long form was developed. These datasets were selected to obtain a sample of children with a diverse range of conditions and different stressors with which to create the SF. Each child was included in only one of the cohorts and each study followed similar processes. The validation cohort (*n* = 49), collected to validate the long-form CLCF, were children not on intravenous antibiotic treatment (IVAT) attending CF specialist clinics in Liverpool, London, and Leeds. The sensitivity cohort (*n* = 32) comprised of well and unwell children with pulmonary exacerbations recruited from a specialist clinic in Liverpool and several linked peripheral sites. The third cohort (*n* = 54) attended one of 27 CF clinics across the UK (excluding Northern Ireland) and completed the long-form CLCF as part of the HIVAT study, all of whom were or had been on IVAT (Glasscoe et al., 2010; Glasscoe et al., 2009).

These data were considered valid for the purposes of this study because whilst the outlook for children with CF is improving, the structure of care remains the same, as does the nature of interactions with healthcare professionals. The items were concerned with the challenges of living with a child with CF which remains a constant concern for caregivers (Davies et al., 2020).

Ethics approval was granted by Liverpool Children’s Medical Research Ethics Committee for the first two cohorts (Ref. 05/Q1502/146). Ethics approval for the HIVAT study was given by the West Midlands Research Ethics Committee (Ref: 08/H1208/11). Informed written caregiver consent, and where appropriate, child assent were obtained before data collection in each cohort.

### 2.2. Measures

#### 2.2.1. Challenge of Living with Cystic Fibrosis (CLCF)

The CLCF is a comprehensive caregiver-reported questionnaire assessing all aspects of the challenge of caring for a child with CF, and a more comprehensive background to its creation is detailed in Glasscoe et al. (2022). The CLCF was developed in a three-stage process encompassing health professional and caregiver viewpoints in a systematic manner and constructed using qualitative methodologies including participatory action research focus groups generating data later refined using cognitive interviews, to produce an abridged final version for general use (see Glasscoe et al., 2022).

The CLCF abridged version consists of two parts; Part A measuring 46 items based on caregiver feedback about family lifestyle, child’s character, challenges to family life, hopes and worries, CF routines and community support, and Part B covering detailed CF treatment information, based on the perceptions of health care professionals. The domains in Part A, which are hidden in the CLCF under more family-friendly headings, are (1) Family Care-Giving Challenges (10 items)—relating to how the family interacts and the practical and emotional impact of CF on family life; Child Challenge (5)—relating to the child’s perceived behaviour and personality; Maintaining CF routines (7)—relating to how the caregivers felt they were managing therapeutic routines; Perceived support (12)—the level of support the caregivers received from stakeholders in their child’s health including their wider social network; Hopes for the Futures (6)—relating to caregivers views of their child’s future, and Worries about Current Health (6)—relating to caregivers worries about their child’s health and the effect it has on them.

All three cohorts completed the long-form validation version of the CLCF, which preceded the final abridged version. This was the version designed to validate the CLCF (Dyer et al., 2010; Patel et al., 2011a) and was subsequently reduced for greater utility as a clinical tool. The items corresponding to the abridged version were then selected and used in this analysis to create the CLCF-SF. See Glasscoe et al. (2022) for both these versions of the CLCF.

#### 2.2.2. Beck Depression Inventory (BDI)

Depression was reported by parents using the second edition of the Beck Depression Inventory (BDI-II) (Beck et al., 1996). This is a 21-item self-report instrument for measuring the severity of depression in adults and adolescents aged 13 years and older. The BDI-II is a widely used measure (e.g. Glasscoe et al., 2007) that has been extensively validated and is reported to have good internal consistency and discriminant validity. A low clinical cut-off of 12/13 was selected to indicate dysphoria (mild depression) and the term dysphoria was preferred as this was a questionnaire-based assessment (Dozois et al., 1998).

#### 2.2.3. The State-Trait Anxiety Inventory (STAI)

The STAI was used to measure trait and state anxiety (Spielberger et al., 1983). It has been used in clinical settings to diagnose anxiety separately from depressive syndromes and as an indicator of caregiver distress in research studies (e.g. Greene et al., 2017). Form Y has 20 items for assessing trait anxiety (how they feel generally) and 20 for state anxiety (how they feel right now). All items are rated on a four-point scale (state ‘Almost Never’ to ‘Almost Always’; trait ‘Not at all’ to ‘Very much so’), higher scores indicating greater anxiety. Construct and concurrent validity of the scale and evidence that it is a sensitive predictor of caregiver distress over time have been demonstrated in several studies (Spielberger, 1989; see https://www.apa.org).

Both the BDI-II and the STAI were administered in the sensitivity cohort but were not included in the main validation study protocol; therefore fewer responses were available for these measures.

#### 2.2.4. Cystic Fibrosis Questionnaire (CFQ-R)

Since its development, the Cystic Fibrosis Questionnaire (CFQ-R) has proven to be a versatile measure of Health-Related QoL that assesses the impact of the disease across a variety of domains of functioning (Quittner et al., 2005, 2012). It is available electronically in eight languages and is used in many CF clinical trials worldwide. It has shown sensitivity to changes in lung function, can predict pulmonary exacerbations, and predicts poor outcomes in patients with CF. We used the six lung function items from the standalone respiratory scale, which were: ‘my child was congested’, ‘my child coughed during the day’, ‘my child had to cough up mucus’, ‘my child wheezed’, ‘my child had trouble breathing’, ‘my child woke up during the night because s/he was coughing’, to provide a measure of a child’s lung function for this study.

### 2.3. Sample size

The sample size (*n* = 135) was deemed sufficient for constructing the SF by the methods used. Little research exists on sample size for the GA method itself as it does depend on the underlying method used to optimise the item selection and the number of items available. Straat et al. (2014) for example, provide minimal sample sizes for an automated Mokken scale extraction using GAs stating that samples from 250 to 1750 are needed, depending on the specific context. However, in this paper, we extract a single scale from a set of items rather than partition existing variables into different scales as is done in Straat et al. (2014).

We had a good set of 46 items from which to select the subset. With too few items all combinations can be created but with poor generalisability, and with too many items the optimal subset may never be reached. We used Cronbach’s alpha as the optimising statistic and the SF has 15 items with a four or five-point Likert scale for every item. Based on an alpha value of 5% and 80% power, and using the formula introduced by Bonett (2002), then with 15 items a sample size of *n* = 135 would be sufficient to estimate a Cronbach’s alpha of between 0.7 and 0.9 to within ±0.1, and in some cases an even narrower width. This sample size was also sufficient to estimate a Pearson correlation coefficient of at least 0.3–0.4 to within ±0.14.

### 2.4. Statistical methods

#### 2.4.1. Item selection procedure using genetic algorithm

The short form was constructed from the 46 scoring items in Part A of the final abridged version of the CLCF (see Glasscoe et al., 2022). These items were considered by the subject matter experts in our team to be key to measuring the challenge of caring for a child with CF. Part B comprised of 144 treatment-related items, which were not applicable in many cases—these items were used as an important part of the validation process described below. A small amount of missing data (∼1.6%) with no observable pattern was replaced with median values for that item.

A GA, written in R (R Core Team, 2017) and similar in form to that presented in Coley (1999), was used to select the subset of items for the CLCF-SF. A detailed description of the GA is found in Appendix A. The specific parameterization of the GA (e.g. population size, number of generations, mutation probability, etc.) is heavily dependent on the specific problem it is used to solve (Coley, 1999). As such the specific parameters were chosen by extensive experimentation, noting that the major effect of a badly specified GA is that it is slow to fit, and unless a local minimum is encountered, differently specified models will find the same solution. Items were chosen according to two opposing criteria, firstly, the test should have as high as possible a value of Cronbach’s alpha, showing the items were internally consistent. To balance this, a penalty function was written to penalise excessively long subset selection. The two criteria push in opposite directions, i.e. the Cronbach’s alpha wants to include as many items as possible while the penalty function wants to remove them. To combine these two criteria the Cronbach’s alpha for a set of items was multiplied by a penalty score, between 0 and 1, to generate the fitness score for a possible solution. This penalty function was expressed as:

$$\text{Penalty}={1-(\text{number of items}‐\text{MAX}(\text{number of items}))}^{3}$$

The polynomial value of the penalty function controls the number of items selected, values 2, 3, 4, and 5 were assessed and the resulting tools were assessed by the experts. It was felt that the third order polynomial provided the most parsimonious solution.

#### 2.4.2. Reliability assessment

The following measures related to the Classical Test Theory (CTT) analysis were calculated using the R package ‘psych’(Revelle, 2017): (i) Cronbach’s alpha, which gives the overall level of internal reliability, and is considered to be ‘excellent’ if ≥0.9, ‘very good’ if ≥0.8, and ‘adequate’ if ≥0.7 (Kline, 2011); (ii) the Cronbach’s alpha if deleted, which gives the Cronbach’s alpha if an item is removed—this highlights the extent to which the given item fits the construct that the rest are measuring; (iii) the scaled mean rating for each item—as some items are on a five-point Likert scale and others are on a four-point Likert scale, the mean rating for each item required standardisation, and this was done by dividing the mean rating for an item across all ratings by the number of response options giving a value between 0 and 1; (iv) the corrected item total correlation, which is the correlation between each item and the overall scale (if that item were removed), providing a measure of the discrimination of the item. Unidimensionality was assessed using exploratory factor analysis (Streiner & Norman, 2008). Exploratory factor analysis was chosen over confirmatory as we were interested in assessing the loadings on multiple dimensions if they existed, rather than just showing that the data fitted the unidimensional model sufficiently well.

#### 2.4.3. Validity

Several measures were utilised to establish convergent validity. Two of the measures (BDI-II, STAI) had fewer caregiver responses available for these analyses, as explained in the Methods. The BDI-II (Beck et al., 1996) measured caregiver depression and there were 79 cases for which this measure was available. The STAI form Y (Spielberger et al., 1983) measured *state* (current) and *trait* (underlying) anxiety. There were 72 state and 66 trait cases for which this measure was available. Both were expected to correlate moderately well with the CLCF-SF (>0.4). A measure of the respiratory health of the child came from the six lung function items from the CFQ-R (Quittner et al., 2005). A low score indicating poor respiratory condition was expected to correlate negatively with the CLCF-SF (> −0.3).

Convergent validity was assessed using the supplementary data from Part B of the long-form CLCF, providing details (including dose frequency) of all treatments (see Appendix 7 Abridged CLCF version in Glasscoe et al., 2022). Three scales were internally constructed to measure different aspects of treatment management using data from the CLCF long-form (these data were excluded from the item selection procedure for the SF). The first was a simple sum score of the number of treatments the child received using data from item 36 ‘Over the last two weeks how much has your child needed the following treatments to keep him/her well?’ The second and third scales summarised the respondents’ view of their own and their child’s competence to manage treatments using data from item 37 ‘We want to know how hard it has been for you to manage these treatments’ and item 38 ‘How do you think your child has managed these aspects of the CF routine over the last two weeks?’ (each rated as very difficult/somewhat difficult/not at all difficult/does not apply). A Rating Scale Model (RSM), a type of polytomous item response theory (IRT) model (de Ayala, 2009), was chosen to create these latter two scales as this method can account for the missing data associated with children receiving different combinations of treatments. The summary scales were expected to correlate positively (>0.4) with the CLCF-SF score. For the measures intended to assess convergent validity, the results are given as a measure of correlation, using Pearson’s ‘r’ or Spearman’s ‘Rho’ coefficient, depending on whether the distribution of the variables followed a normal distribution or displayed a skewed distribution, respectively. The difficulty scores (point estimates) and their standard errors from the model were also used to compare the perceived caregiver and child’s difficulty in managing the treatments given the caregivers’ differing underlying levels of management ability.

For the assessment of discriminant validity, i.e. the extent to which the score could discriminate between known groups, two related measures were used from the CLCF. Firstly, a measure of the perceived wellness of the child was utilised, grouping the responses to the item in the long-form CLCF asking about an illness over the past 3 months as ‘unwell/mostly unwell/mixture of well and unwell’ versus ‘mostly well/well’. Secondly, a measure of whether the child had been admitted to the hospital or not over the past three months was used. The discriminant validity was assessed by comparing the mean CLCF-SF scores of the grouped categories using independent t-tests.

## 3. Results

Data were available from 135 caregivers who completed the CLCF across the 3 different study cohorts. Most caregivers were female (91%) with an average age of 39 years (*SD* 7.3 years). Cohort 1 comprised of 49 children (24 males and 25 females, mean age 9.1 with *SD* 4.2) for which two dates of birth were missing and in one case both parents responded for the same child. Cohort 2 comprised of 32 children (18 males and 14 females; mean age 7.5 years with *SD* 4.3). Cohort 3 consisted of 54 children (21 males and 33 females; mean age 9.9 years with *SD* 2.9) and included one set of twins, and two siblings from the same family, and in two cases two parents both responded for the same child. Overall the mean age of a child with CF was 9.0 years (*SD* 3.9 years) and 72 (53%) were female.

### 3.1. Item selection

A ‘core’ of three items was selected, i.e. items to appear in every solution, and around which the measure was built; ‘How well do you think you are juggling the demands of CF with the needs of your family?’, ‘How well do you think your family as a whole handles the challenges of CF?’ and ‘How much does the responsibility of looking after a child with CF affect you?’. These items were selected by the subject matter experts to reflect the overall role and underlying challenges experienced by all families and were confirmed as robust choices given they had high loadings on one factor in the exploratory factor analysis. This led to a good overall selection of items from the algorithm that related to the challenges experienced by the caregivers. However, no items were selected by the algorithm from the ‘Perceived support’ domain. After discussion among the research team and to improve content validity (Streiner & Norman, 2008) two items from this domain were included in the final ‘core’ set (items 6 and 7 in Table 1) to reflect the close relationship that families may have with their local pharmacist and the specialist CF team. Given that the genetic algorithm is simply a ‘tool’ for finding good sets of items for a particular purpose, our approach has always been to consider expert judgement when making final decisions on item inclusion. The GA was run from six random starting points and converged on the same solution in all cases; this number was therefore felt to be sufficient for assessing whether we had hit any *local minima* (non-optimal solutions).

**Figure 1.**
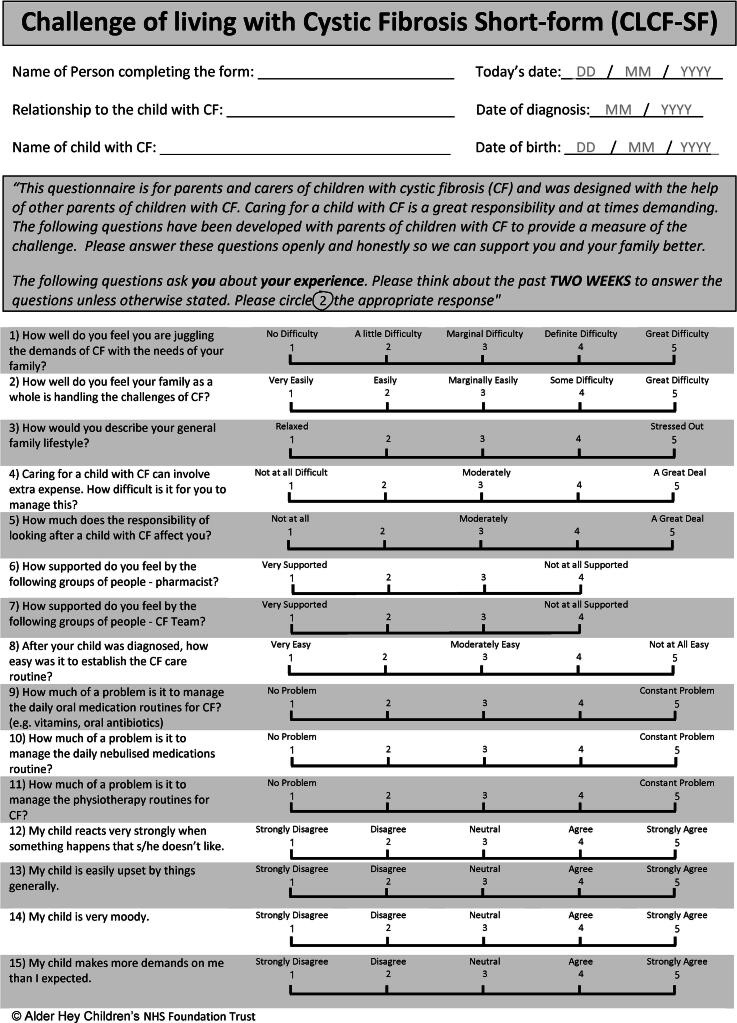
Layout of CLCF-SF.

The CLCF-SF consists of five core items and ten items selected by the GA (Table 1). From these 15 items a summative score was constructed; points are given for each item depending on the Likert scale response (lower scores indicate that families have fewer problems). The minimum possible score was 15 and the maximum was 73. The observed minimum score was 19, maximum 63, mean 39.21, and *SD* 8.62. The distribution of scores was Normally distributed, Shapiro–Wilk (*W* = 0.99, *p* = 0.83). Three parents had scores >2*SD*s above the mean, indicating coping is a substantial challenge. Figure 1 shows the layout of the CLCF-SF.

### 3.2. Reliability assessment

Cronbach’s Alpha for the CLCF-SF items was 0.82 (95% CI 0.78, 0.87) indicating very good internal consistency. The values of Cronbach’s alpha, if deleted (Table 1), show that their inclusion in the measure does not substantively negatively impact the reliability of the instrument. The scaled mean ratings ranged from 0.30 to 0.71 indicating a good spread of items that measured the construct across different levels of CF management ability. The corrected item total correlations (ranging from 0.19 to 0.62) show low to good item correlation with the trait. Exploratory factor analysis (maximum likelihood) on the polychoric correlation matrix of the items gave a second factor eigenvalue of <1 (0.98) suggesting unidimentionality.

### 3.3. Validity assessment

The CLCF-SF score correlated moderately well with the BDI-II (Rho = 0.48, *p* < 0.001), STAI State Anxiety (Rho = 0.41, *p* < 0.001), STAI Trait Anxiety (Rho = 0.43, *p* < 0.001), CFQ-R lung function (Rho = −0.37, *p* = 0.001), caregiver treatment management (*r* = 0.48, *p* < 0.001) and child treatment management (*r* = 0.45, *p* < 0.001), but less well with the sum score of the number of treatments (Rho = 0.19, *p* = 0.035).

**Figure 2.**
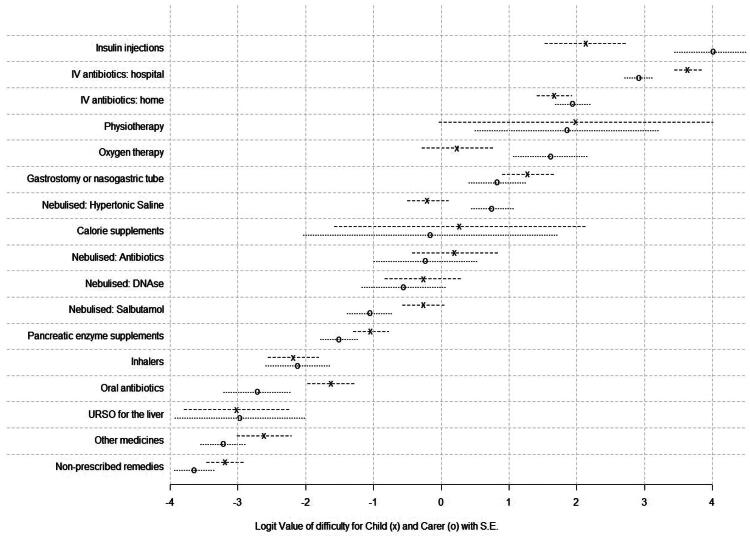
Comparison of child and caregiver treatment management scores from rating scale models.

Figure 2 compares the RSM parameter estimates and standard errors for the item difficulties that made up the treatment management scores for parents/caregivers and children. The perceived difficulty scores (point estimates from the RSM) are represented by a circle for the caregiver and a cross for the child for each treatment listed in the graph, and the lines around the scores on the graph (± the standard error) show the variability in the scores around the point estimate. The items which are perceived as easier to manage by the caregiver or by their child have the lower difficulty values on the logit (log odds) scale while the items which are more difficult to manage have higher values. A larger standard error indicates that a treatment is less consistently rated for management difficulty across caregivers with differing management ability levels. Overall, there was good agreement between the child and caregiver summary scales of competence in managing less invasive treatments, and valid differences were observed when managing the more invasive ones. Caregivers perceived it to be more difficult to manage the administration of insulin injections, oxygen therapy, and nebulised hypertonic saline for caregivers compared to children, whereas hospital administered IV antibiotics were perceived to be more difficult for children than caregivers (Figure 2).

Moderate effect sizes were observed between the CLCF-SF and the health status of the child. Caregivers who reported over the past three months their child was mostly unwell versus well (Mean Difference 5.5, 95% CI 2.5–8.5, *p* < 0.001, Cohen’s *d* = 0.37) and whose child had a hospital admission versus those who had not (Mean Difference 3.6, 95% CI 0.25–6.95, *p* = 0.039, Cohen’s *d* = 0.26) had higher CLCF-SF scores.

## 4. Discussion

We have created a 15-item short-form version of the CLCF for researchers and clinicians to use in assessing the challenges of caring for a child with CF. We used three existing independently collected datasets on caregivers that were unrelated to the original development sample. The final tool retains the ability to measure ‘challenge’ first defined by the caregivers in the original development of the CLCF long form (Glasscoe et al., 2022). As we have shown the challenge of caring for a child with CF goes beyond the administration of difficult treatment regimes, and the 15 CLCF-SF items appropriately reflect themes generated in the original tool that impact family life. Caregivers are at the centre of a network that includes the child with CF, close family, school, hospital, and wider family, all of which make demands on the caregiver.

Despite the considerable progress made over the past 10 years in the treatment options for children with CF, the items selected for the CLCF-SF remain pertinent to the challenge families’ face on a day-to-day basis and are informative in translating their burden of care. Reducing the burden of care remains a high priority research target for people living with CF (Davies et al., 2020), and as such the CLCF-SF tool is as relevant today as it was when the items were first established (Glasscoe et al., 2022). There is an acute need for validated measures across all ages and the CLCF-SF provides a tool for this particularly challenging age group of children.

The utility of the CLCF-SF as a caregiver reported outcome measure is clear and it provides a valid measure of the impact that a child with CF has on the family. The majority of caregivers in our samples were women, indicating that they are likely to be the most instrumental in managing the challenges within the family (Fitzgerald et al., 2018). The CLCF-SF now allows monitoring and implementation of interventions to reduce the challenges faced by caregivers and the child when necessary. The items were determined using clinical judgement and an innovative statistical synthesis to give high levels of the face and content validity and to facilitate ease of use in a clinical setting. The 15-item questionnaire has already been piloted successfully in an intervention study in Scotland to enhance adherence to chest physiotherapy (ScOOp feasibility study, see France et al., 2019a, 2019b) and a second study set in Ireland (Bhatnagar et al., 2023).

A Genetic Algorithm was used to build the tool around a core set of items. This method effectively selected additional items, beyond the core, that measure other aspects of the same trait. This machine learning method for item selection is likely to be more efficient at selecting a relevant set of items than a manual sequential-trial-and-error method. The item characteristics and the strength of the validation evidence for the chosen set of items reinforce the utility of this relatively new and novel procedure.

This study established the reliability of the CLCF-SF and its constituent items individually. The Cronbach’s alpha for the CLCF-SF was 0.82, this is a very good level of internal consistency (Kline, 2011) though not too high, which would suggest some redundant items. Indeed, it is recommended that alpha not exceed 0.9 (Streiner & Norman, 2008). However, it should be noted that the item set was optimised on Cronbach’s alpha and thus this value is likely higher in the tool creation sample than it would be in the population, and further work is needed to confirm this. The values of *Cronbach’s alpha if deleted*, column 3 of Table 1, show that the value of Cronbach’s alpha would increase negligibly if items 6 and 7 were removed but these items were felt to be important by the clinical team and kept in. The *corrected item total correlations*, column 5 of Table 1, show that thirteen items lay above the 0.20 guideline for inclusion (Kline, 1986), items 6 and 7 having values of 0.19 (N.B. these were the highest correlations with the trait of all items from the original *perceived support* domain). This guideline is helpful for identifying weaker items. However, a final decision was taken not to remove any items from the test as the small increase in reliability would be accompanied by a loss of content validity. The two items chosen were the most informative support items and reflected the important functional working partnerships between the family and CF team and pharmacist; within a more medically-oriented view of social support, these relationships should be perceived as working well by caregivers. The day-to-day burden of caregivers may reflect the involvement of other different dimensions of social support but family relations were the most informative. Family relations were addressed by four items from the Family Care Giving Challenge subscale. The addition of ‘How supported do you feel by the following group of people—friends’ may assist a broader social representation but this was not strongly reflected in the analysis, moreover we have seen many instances when perceived family support is lacking, and meaningful in its absence.

Smith et al. (2000) recommend maintaining subscales representation when developing an SF, so the inclusion of the two *perceived support* items seemed reasonable. However, we took the decision not to include items from the *hopes for the future* domain. We did not consider ‘Hopes for the Future’ less psychologically significant with a chronic life-shortening condition in a child whose parents consistently said they lived life one day at a time, quite the reverse. But in the development of the original CLCF questionnaire (Glasscoe et al., 2022), the ‘Hopes and Fears’ title alone triggered an underlying, overwhelming fear in the parent that their child would die before them and was not an issue they wanted to consider. During the original development that section was therefore parsed into two subscales—‘Hopes for the Future’ and ‘Worries about Current Health’. In this study, one item from the latter subscale was included in the SF and we considered this adequate for the purpose of developing an SF framed in terms of parents’ response to the here-and-now, addressing the target content coverage of current ‘burden’. We felt that including further items from a content domain addressing the future in an SF aimed for research use in the current clinical context was unnecessary and might open a sensitive issue that in some cases may need addressing by a clinical psychologist or psychosocial specialist.

The CFCF-SF’s primary validity was demonstrated in terms of the various measures with which it was expected to be correlated, and its discriminative power for known groups, which were expected to have differing levels of challenge. Overall the correlations were higher than anticipated and gave sufficient evidence to suggest that each had an effect on the challenge caregivers were facing. The CLCF-SF score correlated with measures of depression, anxiety, lung function, and parent/child coping with the treatment regime. We opted to use the BDI-II and STAI measures which are both independently validated psychological instruments for the diagnosis of both depression and anxiety, which in our view is as relevant, if not more so, for this population than measures of depression alone. Our assessment of mood disorder here adds to the findings of epidemiological studies (e.g. Quittner et al., 2014) that used the Hospital Anxiety and Depression Scale (Zigmond & Snaith, 1983) (HADS), a freely available screening tool, or in some cases the Centre for Epidemiologic Studies-Depression Scale (CES-D) (Radloff, 1977), an interview-based clinical assessment employed in the diagnosis of depression. Although we have a smaller sample our findings not only concur with the view that mood disorder is prevalent in this population but also on two anxiety components measured separately, they show a strong concordance with the overall challenge involved.

The CLFC-SF score also discriminated between those perceived as ‘well’ and ‘unwell’, and those who had been to hospital recently compared to those who had not. The derived sum score correlated less well with the CLCF-SF, indicating that there is more to consider when taking treatment burden into account than simply adding up the number of treatments given. This was illustrated in the more sophisticated caregiver/child comparisons of treatment burden. We devised a novel scoring method to summarise the treatment management regime (Part B of the CLCF) and it was interesting to note where the caregiver and child views on the difficulty of treatment management differed, caregivers finding it more difficult to manage insulin injections and oxygen therapy in the home for example and the child the hospital-administered IV.

### 4.1. Limitations

Development of the CLCF long form was complex and the publication process was prolonged, which ‘aged’ the datasets over several years. Over this period we have acknowledged that there have been significant developments in CF therapies, but the core care pathways remain unchanged. Whilst outcomes for people with CF have improved, the standard treatment protocols remain key to maintaining good health. The themes explored in the CLCF-SF remain pertinent to the families and their lived experiences. The long form is now published following extensive peer review and formed the framework for the development of the shorter CLCF-SF. Moreover, submission of the CLCF-SF could only progress after the publication of the long form.

Whilst there is reason for greater optimism in this changing landscape, concerns about emerging side-effects have tempered the exclusively positive reports on the effects of modulator therapy. Little is known about how this new treatment will affect younger children and whether similar or different side-effects will be reported. Given that CFTR circulates widely within the brain, the long-term effects of the new modulator (ETI) should be studied and clinical assessment of neuropsychiatric side-effects (e.g. cognitive fogging, difficulties with concentration, headaches, worsening mental health) is warranted. State of the art has progressed, however, challenges (scale, complexity, parent/child acceptance, parent capability, etc.) remain and are captured within this measure. Indeed, new treatment developments may impact coping strategies and post-traumatic growth (Byra et al., 2021), which may moderate the relationship between caregiver burden and adverse mental health outcomes; this changing landscape of CF would be an interesting supplementary study with perceived burden represented by the CLCF as an explanatory variable.

The sample size of 135 collated from three linked studies is a limitation of the study. However because the same clinical investigators oversaw and trained the researchers collecting the data for each study, we were confident that the data had been collected in a consistent manner across studies and therefore could be amalgamated, and moreover, the specific ‘seed’ items used in the GA would remain stable across studies. Our method had sample size constraints in that we used existing data, but the fact that CF is a relatively rare condition also inflicts limitations for future work. The ideal scenario in planning future validation studies would be to run a Monte Carlo simulation study to generate an appropriate sample size now that we have produced initial estimates to use in such simulations from this study.

The range of constructs used to validate the SF could be expanded in future validation studies, for example, we did not include constructs with which very low correlations were expected. Smaller numbers were available for the BDI-II and STAI as these measures were not included in the main validation study and were only included in the HIVAT study after data collection had started due to problems with another measure. The data available did provide sufficient evidence for validation purposes but larger studies using these measures are needed to confirm our findings. We also did not include a comparative caregiver burden measure, such as the Treatment Burden Scale (TBS) of the Cystic Fibrosis Questionnaire-Revised (CFQ-R) (Quittner et al., 2012), as only two of the 3 linked studies had limited data to contribute. Inclusion is recommended in future larger-scale validation studies, as the CFQ-R Treatment Burden Scale has consistently shown that higher treatment burden has been associated with elevated depression scores, and correlated strongly with the CLCF long form (46 items) in a different sample (*n* = 31, *r* = −0.62, *p* < 0.001) (Glasscoe et al., 2022). Here, since a low CFQ-R score indicates a high burden, caregiver challenges increased with a greater treatment burden. Also, data collected on male caregivers would enhance our findings given most caregivers in this study were female (91%) and therefore only give one view from the perspective of the family.

Because we did not have many complete sets of validation measures across the linked datasets, it was difficult to make good comparisons between the SF (15 items) and long-form (46 items) CLCF in this study. However, in future validation work, we will be able to plan and include more validity comparisons of the two measures. In this study, the Cronbach’s Alpha for the CLCF-SF items was 0.82 (95% CI 0.78, 0.87) which compared well with Cronbach’s alpha values on subscales of the CLCF long form of between 0.62 and 0.84 (Glasscoe et al., 2022). Another limitation of our approach was that the dataset was not large enough to report cross-validated values of the validity and reliability statistics. The short-form, therefore, needs to be further validated and refined in larger samples and in other distinct clinical settings. In addition, two of the fifteen items are currently measured on a four-point Likert scale, which we intend to change to a five-point scale in the future for consistency, making the range of scores from 0 to 75. This would require some re-validation to ensure that the adaption of the two items in the measure retains the validity of the tool. This change cannot be validated from our data as although thirteen of the fifteen items were measured on a five-point scale, this was not the case for these two items.

### 4.2. Conclusion

We have created a short-form of the CLCF, which can quantitatively assess the caregiver challenge of living with a child with CF. In addition to this, similar to the long-form CLCF, the short form generates conversations and reflections about the challenge of treatment. The short-form provides clinicians and researchers with a promising tool with much potential clinical and research utility. As a guide to clinical management, the tool will potentially be able to identify caregivers for whom the challenge is perceived as considerable at an early stage and facilitate support for these families. From a research perspective, there is an urgent need for a pragmatic caregiver-reported outcome to reliably measure treatment burden to evaluate the pragmatic impact of any interventions devised to help families. The tool currently has good preliminary validity and external context but requires further validation in distinct clinical settings to ensure that items operate well in all contexts. Additionally, work with stakeholders is needed for establishing meaningful cut-offs and minimally clinically important differences on the scale.

## Data Availability

Data may be made available upon request from author CG. These data are not publicly available due to their containing information that could compromise the privacy of research participants.

## Appendix A —Details of the genetic algorithm

The simple genetic algorithm functioned in the following manner: (1) Initial population generation Each possible solution represents the *n* additional items from the item selection pool to be added to the ‘core’ items to form the questionnaire. These were coded such that each item in the item selection pool was coded as ‘1’ if it were selected and ‘0’ if it were not. This means that a sample solution was represented by a string of 0s and 1s. A population of 100 random strings was created with a bitwise probability of selection of 35%. (2) Fitness assessment The fitness of each string was assessed by computing the Cronbach’s alpha and then multiplying it by the value of the penalty function for the number of items in the solution. (3) Breeding The top ranked 50% of strings, according to fitness, were chosen for ‘breeding’. Twenty-five pairs of strings were bred by choosing a single random location, *via* a uniform distribution, along their length, the *crossover point*, and swapping information from both parents at that point to create two children. (4) Mutation A bitwise mutation probability of 10% was applied to the children, in other words, there was a 10% chance that each 1 in the string could flip to a 0, or *vice versa*. (5) Replacement The generated children joined their parents and replaced the least fit 50% of the initial population to form the new population. (6) Stopping criterion Move back to step 2 and run the algorithm for 250 iterations.

**Table 1.** Internal reliability statistics for the 15 items of the CLCF-SF.

Item text	Domain	Likert scale length	Cronbach’s alpha if deleted	Scaled mean rating	Corrected item total correlation
(1) How well do you think you are juggling the demands of CF with the needs of your family?	Family care-giving challenge	5	0.80	0.46	0.62
(2) How well do you think your family as a whole handles the challenges of CF?	Family care-giving challenge	5	0.81	0.55	0.58
(3) How would you describe your general family lifestyle?	Family care-giving challenge	5	0.81	0.34	0.47
(4) Caring for a child with CF can involve extra expense. How difficult is it for you to manage this?	Family care-giving challenge	5	0.82	0.56	0.38
(5) How much does the responsibility of looking after a child with CF affect you?	Worries about current health	5	0.80	0.71	0.57
(6) How supported do you feel by the following groups of people—pharmacist?	Perceived support	4	0.83	0.44	0.19
(7) How supported do you feel by the following groups of people—CF Team?	Perceived support	4	0.83	0.31	0.19
(8) After your child was diagnosed how easy was it to establish the CF care routine?	Maintaining CF routines	5	0.82	0.56	0.38
(9) How much of a problem is it to manage the daily oral medication routines for CF? (e.g. vitamins, oral antibiotics)	Maintaining CF routines	5	0.82	0.51	0.41
(10) How much of a problem is it to manage the daily nebulised medications routine?	Maintaining CF routines	5	0.81	0.54	0.45
(11) How much of a problem is it to manage the physiotherapy routines for CF?	Maintaining CF routines	5	0.81	0.56	0.55
(12) My child reacts very strongly when something happens that s/he doesn’t like.	Child challenge	5	0.81	0.59	0.54
(13) My child is easily upset by things generally.	Child challenge	5	0.82	0.36	0.39
(14) My child is very moody.	Child challenge	5	0.81	0.30	0.51
(15) My child makes more demands on me than I expected	Child challenge	5	0.82	0.47	0.36

Shaded cells = core items; white cells = items selected by the GA; *n* = 135 families.
